# Yeast aconitase mitochondrial import is modulated by interactions of its C and N terminal domains and Ssa1/2 (Hsp70)

**DOI:** 10.1038/s41598-018-24068-w

**Published:** 2018-04-12

**Authors:** Reut Ben-Menachem, Katherine Wang, Orly Marcu, Zhang Yu, Teck Kwang Lim, Qingsong Lin, Ora Schueler- Furman, Ophry Pines

**Affiliations:** 1 Department of Microbiology and Molecular Genetics, IMRIC, Faculty of Medicine, Hebrew University, Jerusalem, Israel; 2 CREATE-NUS-HUJ Program and the Department of Microbiology, Yong Loo Lin School of Medicine, National University of Singapore, Singapore, Singapore; 3 Department of Biological Sciences, National University of Singapore, Singapore, Singapore

## Abstract

Molecules of single proteins, echoforms, can be distributed between two (or more) subcellular locations, a phenomenon which we refer to as dual targeting or dual localization. The yeast aconitase gene *ACO1* (778 amino acids), encodes a single translation product that is nonetheless dual localized to the cytosol and mitochondria by a reverse translocation mechanism. The solved crystal structure of aconitase isolated from porcine heart mitochondria shows that it has four domains. The first three tightly associated N-terminal domains are tethered to the larger C-terminal fourth domain (C-terminal amino acids 517–778). We have previously shown that the aconitase C terminal domain constitutes an independent dual targeting signal when fused to mitochondria-targeted passenger-proteins. We show that the aconitase N and C-terminal domains interact and that this interaction is important for efficient aconitase post translational import into mitochondria and for aconitase dual targeting (relative levels of aconitase echoforms). Our results suggest a “chaperone-like function” of the C terminal domain towards the N terminal domains which can be modulated by Ssa1/2 (cytosolic Hsp70).

## Introduction

Molecules of one protein can be located in several subcellular locations, a phenomenon termed dual targeting or dual localization. These identical or nearly identical forms of proteins, localized to different subcellular compartments are termed echoforms or echoproteins (to distinguish them from isoforms/isoproteins)^1,2^. Dual targeting has been shown to be highly abundant and we now estimate that in yeast, one-third of the mitochondrial proteome is dual-targeted^3^. Aconitase [citrate (isocitrate) hydroxylase] of yeast is encoded by a nuclear gene, *ACO1* (778 amino acids)^4^, which gives rise to a single translation product that distributes between mitochondria and the cytosol^5,6^. The active site of aconitase involves an iron-sulfur cluster^7^, and the solved crystal structure of aconitase isolated from porcine heart mitochondria shows that it folds into four domains. The first three tightly associated N-terminal domains form a shallow depression where they adjoin near the center of the molecule. The iron-sulfur cluster is ligated to three cysteines of the third domain. The fourth C-terminal domain, which is larger, is associated with the first three by an extended polypeptide chain segment^8^. In mitochondria, aconitase is an enzyme of the tricarboxylic acid cycle (citrate to isocitrate conversion) and stabilizes mitochondrial DNA^9,10^, while in the yeast cytosol it participates in the glyoxylate shunt^5,8,11^. The glyoxylate pathway allows fungal and plant cells to convert two-carbon compounds into four-carbon organic compounds, thereby enabling them to grow on acetate, ethanol or oleate as their exclusive carbon source^12^. The cytosolic population of aconitase is very small (∼5%), a situation that has been called “eclipsed distribution”. In this situation, the predominant quantity of one echoform in a certain subcellular compartment masks detection of small amounts of the other echoform localized elsewhere in the cell. The cytosolic detection of aconitase, in this case, had to be obtained by alternative approaches^5,6,13,14^. In yeast, the dual targeting mechanism of aconitase, fumarase and other proteins, occurs by a mechanism of reverse translocation: the N-terminal mitochondrial targeting sequence (MTS) of all protein molecules is translocated across the mitochondrial membranes and then it is cleaved off by the mitochondrial processing peptidase (MPP). Following cleavage of the MTS, a subpopulation of the protein molecules undergoes reverse translocation back into the cytosol^14–16^.

The C-terminal domain of aconitase (amino acids 517–778) is required for its dual targeting. Fusion of this C-terminal domain to mitochondria-targeted passenger-proteins, such as orotidine-5′-phosphate decarboxylase and dihydrofolate-reductase, conferred on them eclipsed dual localization. Thus, the aconitase C-terminal domain serves as an “independent signal” which is on the one hand necessary and sufficient for dual targeting on the other^17^.

In this study, we show that the aconitase C and N terminal domains interact, that this interaction is important for efficient aconitase post translational import into mitochondria and for aconitase dual targeting (relative levels of aconitase echoforms), and that the interaction between the aconitase C and N-terminal domains can be modulated by Ssa1/2 (cytosolic Hsp70).

## Results

### The N and C terminal domains of Aco1 interact

Preliminary experiments using a yeast two hybrid (Y2H) system suggested that there is a possible interaction between the C and N terminal domains of aconitase^18^. To learn about the nature of this interaction we generated a homology structural model of the structure of yeast aconitase based on the solved structure of bovine aconitase (see Methods). This model suggested that the last 12 C-terminal residues of aconitase (768–778) form an α helix which enhances the interaction of the C-terminal and N terminal domains of the protein^3^. We went on to design a mutant, Aco1-Δ6, which is deleted for the C-terminal six amino acids (amino acids 773–778) and found that this mutant lost its dual distribution, making it an exclusive mitochondrial protein, which is, nevertheless, fully processed and enzymatically active^17^.

**Figure 1:**
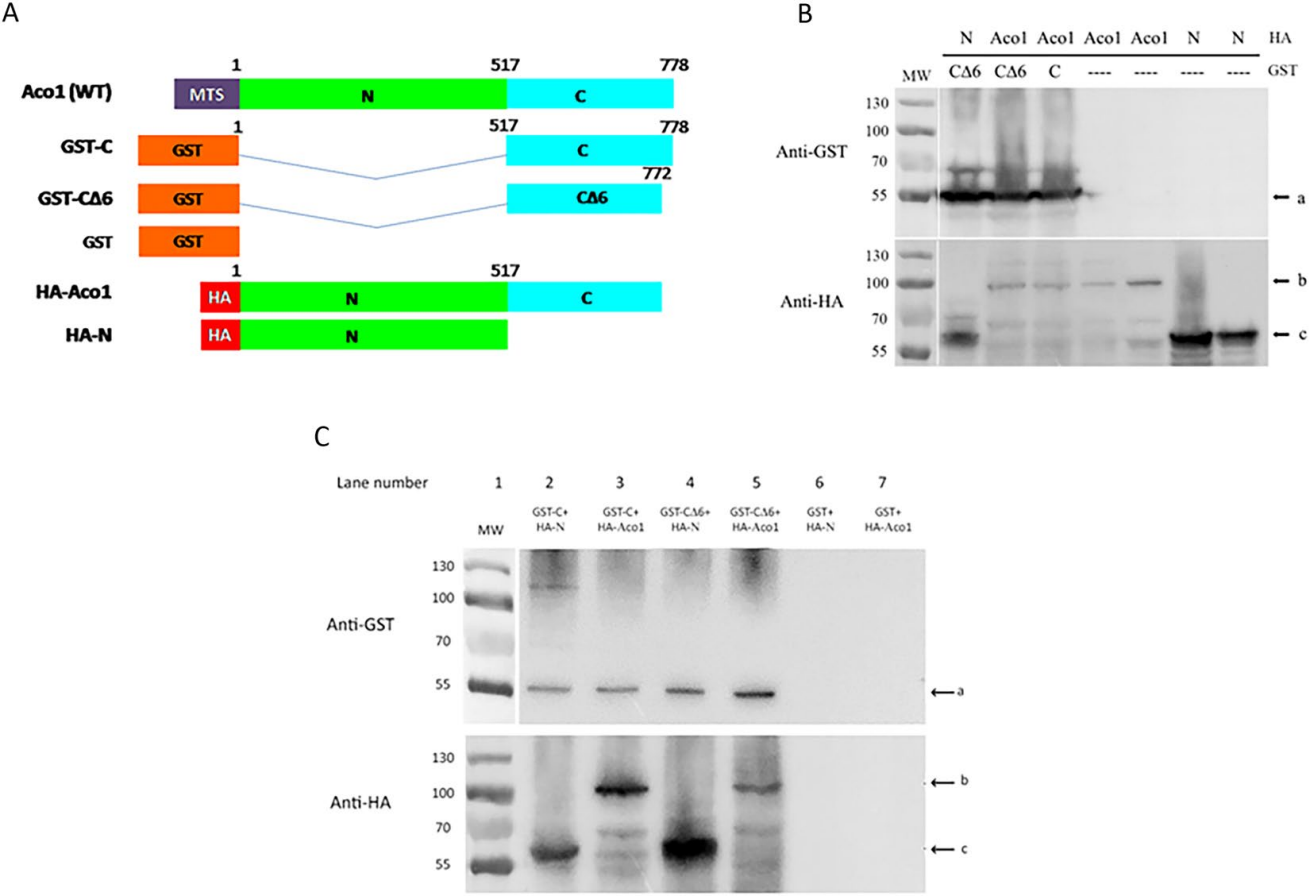
The Aco1 C-terminal and N-terminal domains interact *in vivo*. (**A**) Schematic representation of aconitase GST and HA fusion proteins. N terminal domains, green; C terminal domain, light blue; MTS, purple; GST tag, orange; HA tag, red. (**B**) Aco1 C and N terminal fusions are expressed in yeast. Total cell lysates of yeast cultures expressing the indicated GST and HA fusions were subjected to Western blot analysis using either anti–GST or anti–HA antiserum. Arrows indicate bands of GST-C or GST-C∆6 fusions (“a”), HA-Aco1 fusions (“b”) and HA-N fusions (“c”). (**C**) GST-Aco1-C (C terminal domain) can pull-down full length aconitase and the N-terminal domains. Lysates of yeast cells expressing the indicated fusion proteins were incubated with glutathione beads which were subsequently washed and resuspended in SDS containing loading buffer. Samples were subjected to Western blot analysis using the indicated antisera. Arrows indicate bands of GST-C or GST-C∆6 fusions (“a”), HA-Aco1 fusions (“b”) and HA-N fusions (“c”).

To directly examine if the C and N terminal domains interact, we performed co-precipitation of separately expressed aconitase C-terminal and N-terminal domain polypeptides. GST (Glutathione S Transferase) was fused to the unmutated C-terminal terminal domain (GST-C), or to the C-terminal domain lacking the last 6 amino acids (GST-CΔ6), while the HA (hemagglutinin) tag was fused to the N terminal domains (HA-N) or to the full Aco1 mature protein sequence, lacking its MTS (HA-Aco1) (Fig. 1A). As shown in Fig. 1B, we were able to detect these fusion proteins in total yeast lysates by Western blotting using either anti-GST (top panel, arrow “a”) or anti-HA antiserum (bottom panel, arrows “b” and “c”). Figure 1C shows Western blots of GST pull downs of yeast lysates expressing combinations of the fusion proteins referred to in Fig. 1A. The top panel shows, as expected, that we can detect the GST fusion proteins (top panel, arrow “a”) while the bottom panel shows that the C terminal domain (GST-C) can pull down the N terminal domains (HA-N, lane 2, arrow “c”). The C terminal domain (GST-C) can also pull down the full Aco1 protein (HA-Aco1, lane 3, arrow “b”). This pull-down ability of the C-terminus is also true for GST-CΔ6 (Aco1 lacking the last 6 amino acids, lanes 4 and 5) suggesting that the C-terminal helix is not a requirement for interaction between the C and the N terminal domains.

**Figure 2:**
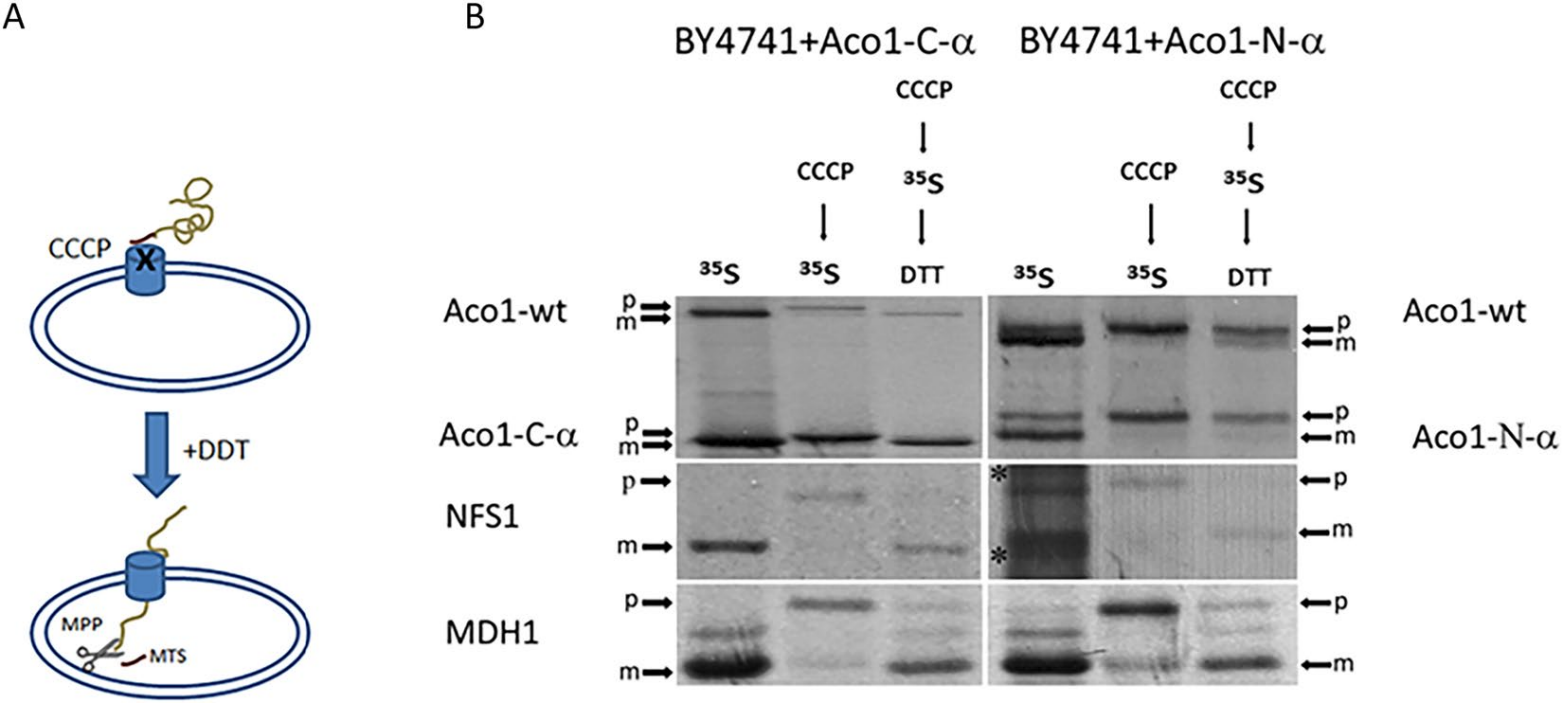
(**A**) Illustration of post-translational import experiment. MTS, brown. (**B**) Aco1-N and endogenously co-expressed aconitase exhibit a defect in mitochondrial import. Yeast strains harboring plasmids encoding the indicated proteins were induced in galactose medium and pulse labelled with [^35^S] methionine–cysteine and CCCP. An aliquot of these cultures was further incubated with DTT in order to restore post-translational import of proteins into mitochondria in the presence of cold methionine–cysteine. Total cell extracts were immunoprecipitated with Aco1, NFS1 and MDH1 antisera and SDS-PAGE followed by autoradiography was performed. Mature, m; Precursor, p.

We have previously shown that a polypeptide containing the first three N-terminal domains of aconitase, displays a defect in post-translational import, whereas, the C-terminal domain on its own displays efficient post-translational import^3^. Thus, a possible problem in post-translational import may originate from the N terminal domain polypeptide and in particular this may be affected by its interaction with the C terminal domain. We examined the post-translational import of C and N terminal domains but this time we made an effort to detect what happens to endogenously expressed aconitase. Yeast cells expressing Aco1-N or Aco1-C (tagged with α) were labelled for 15 minutes with ^35^S-Methionine and CCCP (Carbonyl cyanide m-chlorophenylhydrazone), a membrane potential uncoupler. The CCCP/^35^S-Methionine treated cells were further incubated in the presence of DTT (Dithiothreitol) to inactivate the CCCP and restore membrane potential, while excess cold methionine and cysteine were added to block further labelling. The first treatment causes the accumulation of precursors (“p” band) in the absence of membrane potential (CCCP- > ^35^S, middle lanes) which is required for import. The second treatment checks import of accumulated precursors post-translationally, after restoration of the membrane potential (CCCP- > ^35^S- > DTT, right lanes) [See illustration in Fig. 2A]. Cell extracts, following immunoprecipitation, were analysed by SDS-PAGE and autoradiography.

As shown in Fig. 2B, Aco1-C and endogenously co-expressed aconitase (top left panel, two lower and two higher arrows respectively) are efficiently imported post-translationally (top left panel, only “m”, mature forms are detected in the right lane), while, Aco1-N and endogenously co-expressed aconitase (top right panel, two lower and two higher arrows respectively) exhibit a clear defect in mitochondrial import (top right panel, both “m” and “p” forms appear in the right lane). To rule out the possibility that expression of Aco1-N blocks the import apparatus of mitochondria, and thereby impedes import of all mitochondrial destined precursors, we also examined import of unrelated proteins. As shown in the bottom panels of Fig. 2B, Nfs1 and Mdh1 are efficiently processed when co-expressed with either Aco1-C or Aco1-N. These results suggest that the Aco1-N terminal domain, can associate with endogenously expressed aconitase and affect its mitochondrial targeting, thus provides evidence for the interaction of aconitase domains *in vivo*.

### Disruption of a predicted C/N salt bridge slows down aconitase import into mitochondria

**Figure 3:**
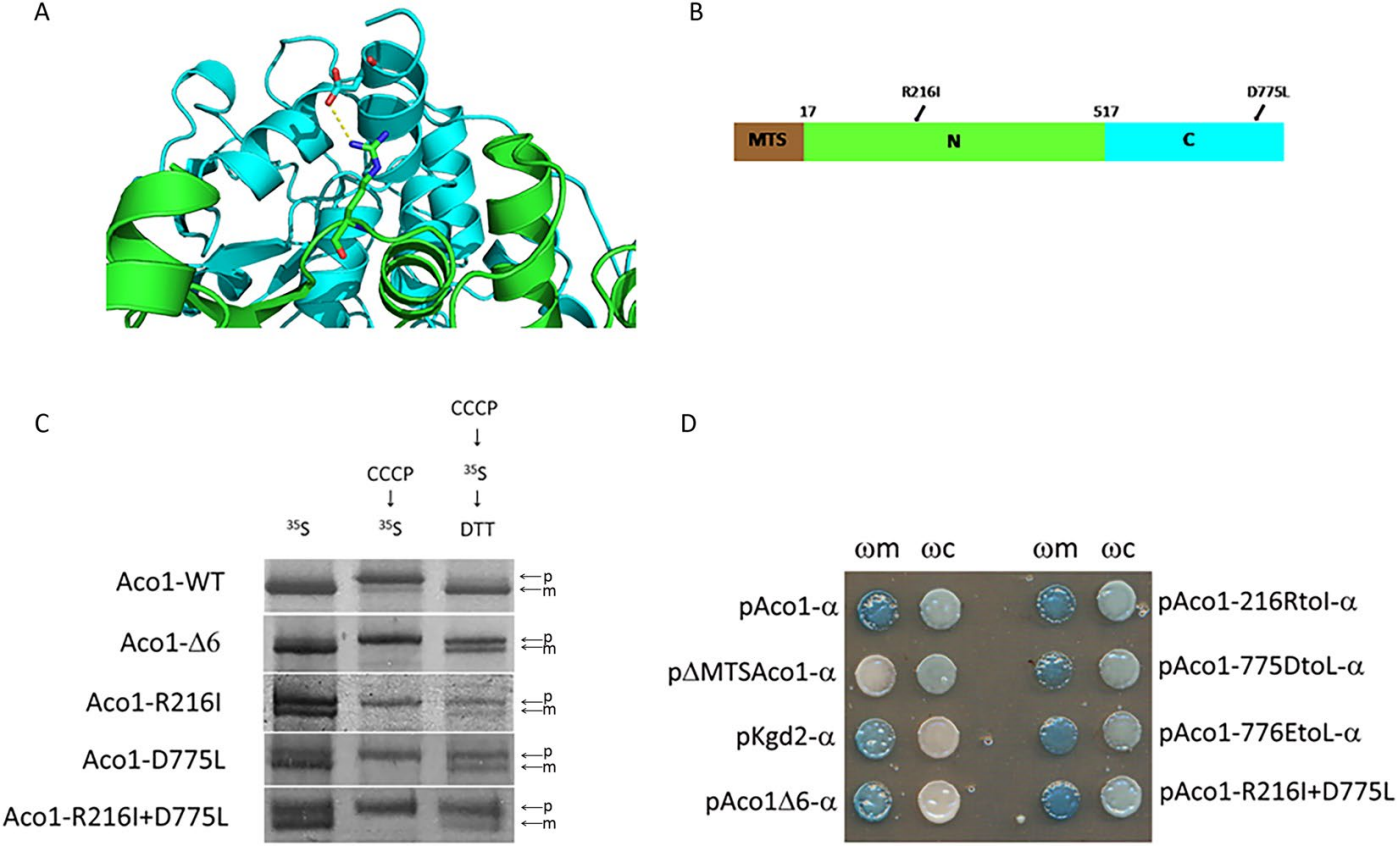
Post-translational import of mutant Aco1 and N/C domains. (**A**) The predicted salt bridge between Aco1 N and C terminal domains, based on a model generated by the I-TASSER software. N terminal domains, green; C terminal domain, light blue; Predicted salt bridge, yellow. (**B**) Schematic illustration of aconitase predicted salt-bridge mutations. MTS, brown; N terminal domain with the R216I mutation, green; C terminal domain with the D775L mutation, light blue. (**C**) Single mutations in the predicted salt bridge affect import into mitochondria. Wild-type yeast cultures harboring the indicated plasmids were induced in galactose medium and labelled with [^35^S] methionine in presence of CCCP which blocks import (left lanes). Following labelling, aliquots of these cultures were chased with excess cold methionine/cysteine in the presence of DTT for an additional 10 min (right lanes). Cell lysates were than immunoprecipitated with aconitase antiserum and analyzed by SDS-PAGE followed by autoradiography. Precursor, p; Mature, m. (**D**) Alpha complementation of the predicted salt bridge mutants. Yeast cultures co-expressing cytosolic ω (ωc) or mitochondrial ω (ωm) together with various α-fused proteins were grown on galactose medium containing X-gal. Blue colonies indicate α fragments that are associated with the ω fragments. Controls: pKgd2- α (dihydrolipoyl transsuccinylase 2) as a mitochondrial marker; pΔMTSAco1- α (Aconitase lacking mitochondrial targeting signal) as cytosolic marker.

Based on the structural model of aconitase, we looked for mutations that would affect the interaction between its N and C domains. We chose to disrupt a predicted salt bridge between arginine 216 (in the N terminal domain) and aspartate 775 (in the C terminal domain; Fig. 3A and B). Accordingly, mutations were introduced that convert both these residues into hydrophobic residues, isoleucine and leucine respectively (Aco1-R216I and Aco1-D775L). We predicted that a single mutation would disrupt the salt bridge, while a double mutant may generate an alternative, hydrophobic interaction (between isoleucine and leucine) that could supposedly suppress this effect. To examine the effect of these mutations on import into mitochondria, post-translational labelling experiments were performed (Fig. 3C) as described earlier (see Fig. 2B). As shown in the right lanes of Fig. 3C, wild type Aco1 is fully processed (“m” band) indicating full post translational import into mitochondria, whereas the mutants exhibit partial processing (see both “p” and “m” bands). Thus, each one of these mutations had a significant effect on post-translational import (Fig. 3C, right lanes). However, there was no compensatory effect of the double mutant (R216I-D775L) which is most probably due to the fact that the volume between these two residues in the model does not allow tight hydrophobic packing without additional structural reorganization. It is worth pointing out that the Aco1Δ6 derivative of aconitase referred to in the introduction also exhibits post-translational delay in import, as will be discussed in more detail below. We then asked whether dual distribution ability of these mutants is retained. Since aconitase is eclipsed distributed with minute amounts in the cytosolic fraction, we had to use the sensitive but qualitative α-complementation assay. The α-complementation assay employs the capability of two peptide fragments of β-galactosidase (designated ω-993 amino acids; α-77 amino acids) to assemble *in vivo* into a complex that displays enzymatic activity, as has been shown in prokaryotic and eukaryotic cells^19–21^. The large quantity in the predominant location does not generate a signal on its own and so does not obscure the signal from the eclipsed protein in the second location. Aconitase mutant proteins described above were expressed under the GAL1 promoter while fused at their C terminus to the α fragment. These fusion proteins were co-expressed in wild-type yeast cells together with either mitochondrial or cytosolic ω fragments (ωm or ωc, respectively). Blue colonies indicate cells with co-expression of aconitase-α mutant proteins together with ωm or ωc when grown on plates containing X-gal. Using this qualitative assay, we found that the single mutations Aco1-R216I and Aco1-D775L, and the double mutation Aco1-R216I/Aco1-D775L exhibit a dual localization phenotype (Fig. 3D). This is in contrast to the Aco1Δ6 and Kgd2 phenotypes which are exclusively mitochondrial. Thus, while these results suggest that the interaction between the C and N terminal domains are important for efficient import, their effects on dual targeting remains elusive.

### Ssa1 binds Aco1 and can separately bind its N and C terminal domains

**Figure 4:**
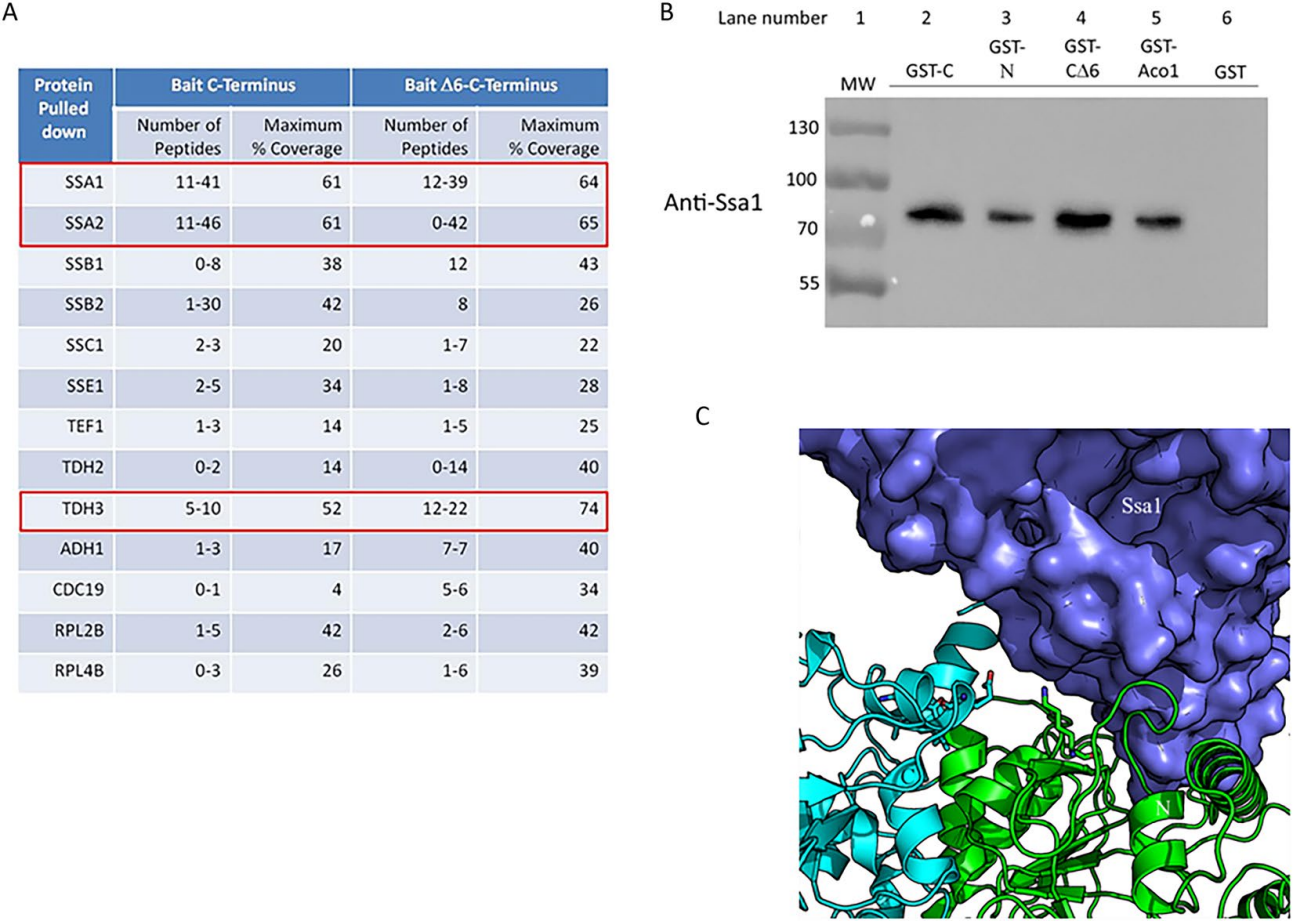
Ssa1/Hsp70 binds Aco1 and its C and N terminal domains. (**A**) Identification of proteins associated with Aco1-C by LC-MS/MS. GST-C or GST-CΔ6, expressed in yeast, were pulled down from lysates using glutathione beads. Proteins were released from the beads and digested with trypsin in solution or run on PAGE followed by in-gel digestion with trypsin. The peptides associated with the Aco1 C-terminus were identified by LC-MS/MS. The number of peptides and coverage of the protein sequence in each case is presented. (**B**) Full length aconitase (GST-Aco1), GST-Aco1-C and GST-Aco1-N can pull-down Ssa1 from yeast lysates. Lysates of yeast cells expressing the indicated fusion proteins were incubated with glutathione sepharose beads, which were subsequently washed and resuspended in SDS containing loading buffer. Samples were subjected to Western blot analysis using anti-Ssa1 antiserum (GenScript. Lot number-642114S01/P20011507). (**C**) Structural model of the Aco1 - Ssa1 complex. The predicted complex structure was created by docking the structural models of Ssa1 and Aco1 (generated using I-Tasser) using PatchDock and then refining it using RosettaDock. Aco1-N, light green; Aco1-C, light blue; Ssa1, purple.

Are cytosolic factors involved in the targeting, import and dual localization of aconitase? The structural model of yeast aconitase revealed a solvent-exposed C terminal domain, which suggested that it could bind to a yet unidentified factor in *trans*. With this notion in mind, we set out to determine who binds the aconitase C-terminal domain, since such factors, if discovered, could also affect the N/C domains’ interaction. GST-C or GST-CΔ6 (bait), expressed in yeast, were pulled down from lysates using glutathione beads. After washing the beads thoroughly, enriched proteins were released from the beads and digested with trypsin in solution or run on PAGE followed by in-gel digestion with trypsin. Figure 4A shows the peptides associated with the Aco1-C terminal domain, identified by LC-MS/MS. A number of proteins of the Hsp70 family were identified (Ssa1/2, Ssb1/2, Ssc1, Sse2). Amongst these Ssa1/Ssa2 stood out with the highest number of detected peptides and the highest coverage of the protein sequence. The cytosolic Hsp70/Ssa1–4 family has been implicated in mitochondrial import, in stabilization of folded proteins^22^, and also in the dual targeting of the enzyme fumarase^15^, which shares the same dual targeting mechanism with aconitase. Therefore, Ssa1 was our first choice for further investigation. To verify this interaction, we performed a standard pulldown of different GST-aconitase fusion proteins and checked for the presence of endogenous Ssa1. As shown in Fig. 4B, GST fusions of the C-terminal domain (GST-C, lane 2), N terminal domains (GST-N, lane 3) and the full mature aconitase (GST-Aco1, lane 5), were capable of pulling down Ssa1 from yeast extracts. This is also true for the C-terminal domain lacking the last 6 amino acids (GST-CΔ6, lane 4). Thus, Ssa1 can bind both the N and C terminal domains of aconitase.

**Figure 5:**
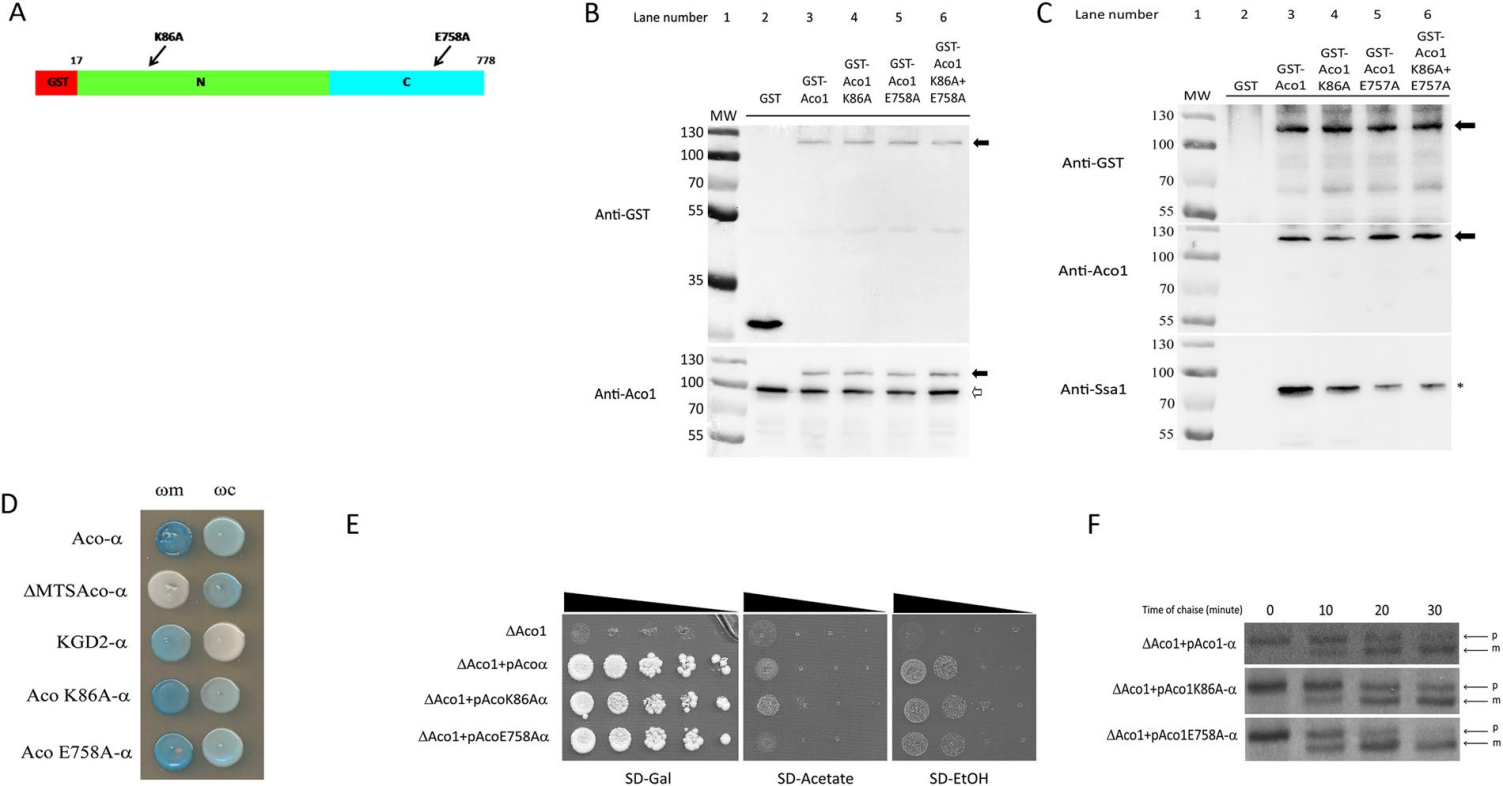
Predicted Aco1 mutations reduce Aco1/Ssa1 binding. (**A**) Schematic representation of GST-Aco1 and mutations causing amino acid substitutions at the predicted Aco1/Ssa1 interface. GST tag, red; N terminal domains with the K86A mutation, green; C terminal domain with the E758A mutation, light blue. (**B**) Expression of GST-aconitase K86A and E758A mutant proteins. Total lysates of yeast cells expressing the indicated fusion proteins were subjected to Western blot analysis using either anti-GST (top panel) or anti-Aco1 (bottom panel). Arrows indicate plasmid encoded aconitase fusions, empty arrows indicates chromosomally encoded aconitase. (**C**) Pull down of Ssa1 by GST and GST-Aco1 mutants. The cell lysates above (B) expressing the indicated GST fusions were pulled down with glutathione sepharose beads and subjected to Western blot analysis using either anti-GST (top panel), anti-Aco1 (middle panel) or anti-Ssa1 (bottom panel) antisera. Arrows indicate the bands of GST-aconitase and its mutants, asterisk indicates bands of Ssa1. (**D**) Alpha complementation assay of aconitase-Ssa1 binding mutants. Yeast WT cells co-expressing cytosolic ω (ωc) or mitochondrial ω (ωm) together with the indicated Aco1-α mutants were grown on galactose medium containing X-gal. Blue colonies indicate alpha fragments that are associated with the ω fragments. Representative controls- pKgd2-α as a mitochondrial marker, pΔMTSAco1-α as a cytosolic marker. (**E**) Aconitase predicted Ssa1 binding mutants show different growth phenotype on acetate and ethanol medium plates. Aco1 KO yeast strains harboring the indicated plasmids were grown at the indicated dilutions on acetate, ethanol and galactose plates. (**F**) Mutations affecting the Aco1-Ssa1 interaction affect the efficiency of aconitase import. Aco1 KO yeast strains harboring the indicated plasmids were induced in galactose medium and pulse labeled with [^35^S] methionine–cysteine for 15 min, followed by a chase (addition of cold methionine–cysteine) for the times indicated. Total cell extracts were immunoprecipitated with aconitase antiserum and SDS-PAGE followed by autoradiography was performed. Precursor, p; Mature, m.

In order to characterize the Aco1-Ssa1 interaction further, we generated a model of the structure of this complex, based on homology models of the individual partners (see Methods). Among the top 20 interaction models generated by PatchDock^23,24^, the first and third model positioned the C terminal (last 12 residues) helix at the interface. These models were further refined with the RosettaDock local refinement algorithm^24^ to generate models with atomistic detail of the interaction (Fig. 4C). Our top-ranking model positions aconitase in the reported substrate binding site of the Ssa1 substrate binding domain [SBD], where also other folded substrates have been shown to bind^22^. Based on manual inspection of aconitase residues found at the proposed interface with Ssa1, K86 and E758 were suggested to contribute significantly to the binding of Aco1 to Ssa1 (Fig. 5A). We chose to introduce mutations at these positions to alanine, to interfere with Aco1-Ssa1 binding. E758 is located within the modelled interface, but does not form any significant interactions with Ssa1. Therefore, an effect of that mutation would suggest a possible role for Ssa1 in breaking the interaction between the C and N terminal domains, while an effect of the K86A mutation would indicate either destabilization of the Ssa1-aconitase interaction, or hindrance of the C and N terminal domains interaction. Figure 5B shows the expression of these mutant aconitases in yeast. Western blotting of lysates using anti-GST (top panel) or anti-Aco1 antiserum (bottom panel) reveals that, while the level of the aconitase fusion proteins was the same in the total lysates (Fig. 5B) and in the GST pull down preparations (Fig. 5C, top two panels), the level of Ssa1 pulled down was clearly reduced in the aconitase mutants (Fig. 5C, compare lane 3 containing unmutated GST-Aco1 with lanes 4, 5 and 6 containing the mutants). As for the mutants, The E758A mutation had a much stronger effect on Ssa1 binding, and its combination with the K86A did not yield any additive effect (Fig. 5C, compare lanes 5 and 6). These results support a specific interaction between Ssa1 and Aco1, which can be diminished by single amino acid substitutions located presumably at the complex interface.

### Consequences of the Ssa1/Aco1 interaction

As discussed above, the proposed roles of the C terminal domain of aconitase are to confer dual targeting and efficient post translational import on the N terminal domains. How then does the interaction of Ssa1 with Aco1 affect these functions? One of the problems in following dual localization of aconitase is that it is eclipsed distributed with a minute amounts in the cytosol. In fact, using the qualitative alpha complementation assay, we detect dual distribution of K86A and E758A aconitase mutants similarly to the wild type (Fig. 5D). Nevertheless, we were able to detect two phenotypes which are consistent with less aconitase remaining in the cytosol; i) The E758A mutant exhibits a slight growth phenotype on ethanol and acetate plates compared to the wild type and the K86A mutant (Fig. 5E, compare row 4 to rows 2 and 3). ii) Employing pulse chase experiments, we find that the E758A mutant is post-translationally imported into mitochondria faster than the wild type and the K86A mutant (Fig. 5F, compare the lower panel to the two above). In this experiment, aconitase mutants were labelled and accumulated in the yeast cytosol by labelling cells with ^35^S-Methionine and CCCP for 15 minutes. Then DTT was added to restore the mitochondrial membrane potential and the ability to import proteins and excess cold methionine and cysteine were added to block further labelling. Aliquots were taken as a function of time, and following immunoprecipitation, were analysed by SDS-PAGE and autoradiography. Thus, these results suggest that disrupting the Ssa1 interaction with Aco1 increases import rates, thereby causing lower levels of aconitase in the cytosol and reducing the growth on acetate and ethanol via the glyoxylate shunt.

### Ssa1 breaks the aconitase C/N domains interaction

**Figure 6:**
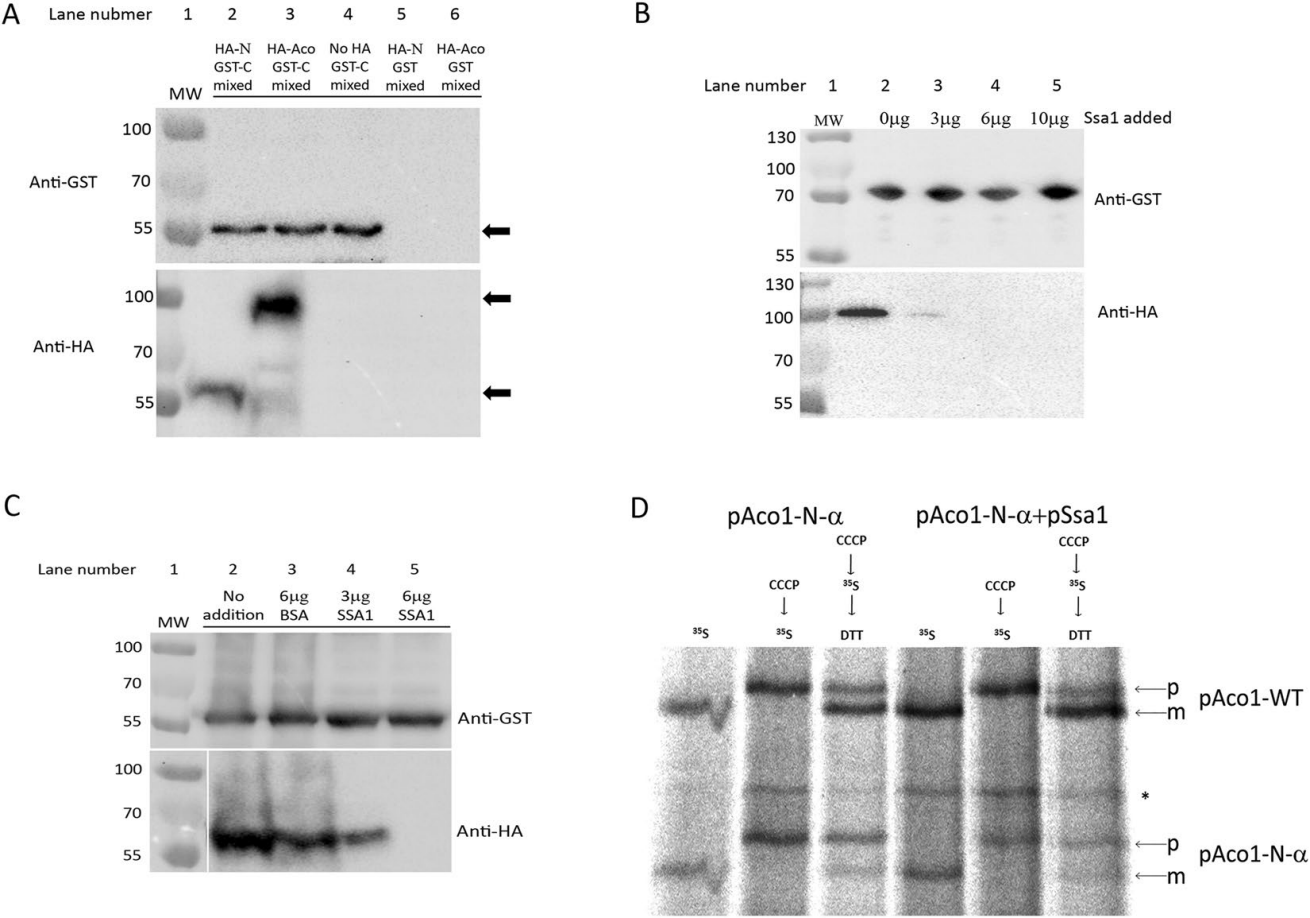
Ssa1 breaks the interaction of Aco1-C with Aco1-N terminal domains. (**A**) Aco1 polypeptides originating from separate lysates form complexes *in vitro*. Separate cell lysates prepared from yeast cells expressing the aconitase C-terminus fused to GST (GST-C) and either Aco1 or Aco1-N fused to HA (HA-Aco1 or HA-N respectively) were mixed and incubated with glutathione sepharose beads. The mixed lysates were pulled down with glutathione sepharose beads and analyzed by Western blotting using GST and HA antisera. Arrows indicate the bands of GST-C (top panel), HA-Aco1 (bottom panel, top arrow) and HA-N (bottom panel, bottom arrow). (**B**) Ssa1 reduces the amount of HA-Aco1 pulled down by GST-C. Separate cell lysates prepared from yeast cells expressing GST-C and HA-Aco1 were mixed in the presence of different amounts of purified Ssa1 (GenScript, Lot number- U9459BB190S01/P20011602). Glutathione sepharose beads were used to pull down the GST fusion proteins and associated proteins which was analysed by Western blotting using GST and HA antisera. (**C**) Ssa1 reduces the amount of HA-N pulled down by GST-C. Separate cell lysates prepared from yeast cells expressing GST-C and HA-N were analyzed as described in (**B**). (**D**) Aco1-N and endogenously co-expressed aconitase import efficiency in the absence or presence of over-expressed Ssa1. Yeast strains harboring plasmids encoding the indicated proteins were induced in galactose medium and pulse labeled with [^35^S] methionine–cysteine and CCCP. An aliquot of these cultures was further incubated with DTT in order to restore post-translational import of proteins into mitochondria in the presence of cold methionine–cysteine. Total cell extracts were immunoprecipitated with Aco1, antisera and SDS-PAGE followed by autoradiography was performed. Precursor, p; Mature, m. Asterisk indicates nonspecific bands. This experiment was repeated three times. A significant difference in the standard error was found between the ratios of mature (m) versus total [precursor (p) + mature (m)] forms of chromosomally encoded wild type aconitase with and without Ssa1 overexpression (lanes 3 and 6).

We hypothesized, based on the data above, that Ssa1 may affect the interaction of the aconitase C terminal domain (Aco1-C) with the N terminal domains, thereby affecting dual targeting and post translational import rates. Given the strong affinity between the C and N terminal domains of Aco1 (Fig. 1C), we attempted to reconstitute this interaction *in vitro*. Lysates of yeast cells expressing either GST-C, HA-N, HA-Aco1 or various combinations of these, were mixed and incubated for 2 hours. The mixed lysates were treated with glutathione beads and pulled down as described in previous sections. As shown in Fig. 6A, GST-C can pull down both HA-N as well as HA-Aco1 (lanes 2 and 3 bottom panel respectively), indicating that Aco1 polypeptides originating from separate lysates form complexes *in vitro*. We then went on to see whether Ssa1 can affect this complex formation *in vitro*. Purified Ssa1 was added to mixed lysates and these were incubated for two hours prior to GST pull down experiments as described above. As shown in Fig. 6B, adding increasing amounts of Ssa1 reduced the amount of HA-Aco1 pulled down with GST-C (top panel). Figure 6C shows that the same is true for GST-C/HA-N; adding increasing amounts of Ssa1 reduced the amount of HA-N pulled down with GST-C (bottom panel) while adding an unrelated control protein, BSA, had no effect.

To understand the importance of this effect of Ssa1, we pulse-labelled wild type cells (as already described) expressing the aconitase N-terminal domains in the absence or presence of overexpressed Ssa1. Our notion was that if the C/N interaction is indeed important for import, expressing Ssa1 will break this interaction and influence the import of the wild type protein. Indeed, as shown in Fig. 6D, overexpressing Ssa1 reduces the inhibitory effect of the N terminal domain on the wild type aconitase import. These results support our hypothesis that Ssa1 breaks the Aco1-C/Aco1-N interaction, which in turn affects the ability of the C terminal domain to confer on aconitase efficient post translational import and dual localization.

## Discussion

To fully grasp the interpretation of our current results we must first briefly summarize what is known about aconitase dual targeting. The yeast enzyme aconitase is distributed between the cytosol and mitochondria by a reverse translocation mechanism. The mature cytosolic and mitochondrial aconitase echoforms are identical products and are targeted to and processed in mitochondria (proteolytic removal of the MTS) before final distribution. Thus, the aconitase amino terminus is processed in mitochondria, and then “returns” to the cytosol. The aconitase fourth, C-terminal domain serves as an “independent signal” which is on the one hand necessary and sufficient for dual targeting on the other. The C-terminal domain fused to mitochondria-targeted passenger proteins conferred dual localization on them^17^. What is the precise mechanism of aconitase dual targeting and how does the C-terminal signal work? We suggest two scenarios or a combination of these: 1) Aconitase and its C-terminal domain fold and block full import of a subset of molecules into mitochondria which are consequently reverse translocated, and 2) Aconitase and its C-terminal domain bind cytosolic proteins which affect its full anterograde import and cause reverse translocation. In this regard a major technical challenge in the analysis of aconitase dual targeting is that it is eclipsed distributed. We have previously shown that protein folding and translocation efficiency affect dual targeting of aconitase^14^: Aconitase import into mitochondria was slowed down by either (i) disturbing the activity of the TOM complex, (ii) lowering the growth temperature, or (iii) introducing mutations in the MTS (Mitochondrial Targeting Sequence) thereby reducing mitochondrial targeting efficiency. In all these cases, cytosolic presence of aconitase was increased, supporting our model that slowing down translocation provides a prolonged opportunity for the protein to fold or bind cytosolic proteins, thereby promoting reverse translocation. Yeast fumarase which has been studied in more detail, is an example of this type of mechanism, and the above findings are also true for this enzyme^15,16^.

In this study we provide insight into two novel aspects of aconitase mitochondrial targeting: the functional interaction of the N terminal domains with the C-terminal domain, and the effect of the yeast cytosolic Hsp70, Ssa1/2, on this interaction. The first aspect is demonstrated by several observations: i) The C and N terminal domains interact *in vivo* (Fig. 1), ii) the C and N terminal domains interact *in vitro* (Fig. 6A), and iii) mutations at the C-N interface affect this interaction, to slow post-translational import of these mutant aconitases (Fig. 3C). The relationship between the N and C terminal domains is interesting: a polypeptide containing the N terminal domains is imported slowly, moreover, upon overexpression it imposes slow import on the endogenous aconitase (by binding to full aconitase). The protein is imported efficiently when the N-terminal domains are attached to the C terminal domain,. These results suggest a “chaperone-like function” of the C terminal domain towards the N terminal domains. The second aspect of aconitase dual targeting and of the C-N terminal domains’ interaction is revealed by Ssa1: i) Ssa1/2 was initially identified by a screen based on an aconitase C-terminus bait and detection by mass spectrometry (Fig. 4A), ii) co-precipitation shows binding of Ssa1 to the aconitase C and N terminal domains and to the full protein (Fig. 4B), iii) Mutations in aconitase, at the predicted Aco1/Ssa1 interface, reduce the binding of these two proteins (Fig. 5C), iv) One of these mutant aconitases exhibits higher post-translational import rate (Fig. 5F), vii) *In vitro* Ssa1 can break the interaction between the C terminal domain and full aconitase and between the C terminal and the N-terminal domains of aconitase (Fig. 6B,C), and finally viii) Overexpression of Ssa1 affects the post translational import of Aco-N terminal domains and its influence on the endogenous aconitase (Fig. 6D).

**Figure 7:**
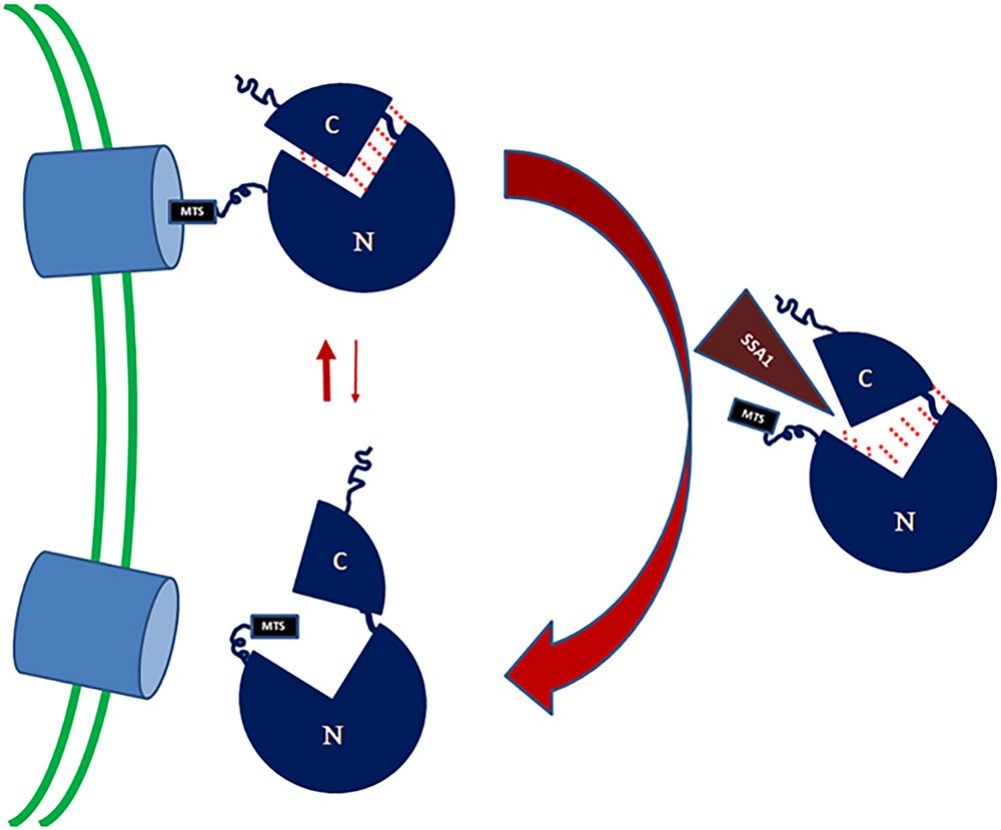
Model of the aconitase targeting mechanism in yeast. The aconitase N-terminal domains which are first translated on the ribosome, are initially poorly translocated into mitochondria. Upon translation of the whole protein, the C-terminal domain can then chaperone the N-terminal domains into a post translational mode of import. Ssa1/2 can affect targeting by binding aconitase and decreasing the interaction between the N and C terminal domains, thereby slowing import and causing more aconitase to end up in the cytosol. **C-** C terminal domain; **N**- N terminal domains; **MTS**- mitochondrial targeting signal.

Based on these results, we now can update our model of aconitase dual targeting (Fig. 7): The aconitase N terminal domains, first translated on the ribosome, is poorly co-translationally translocated into mitochondria until the C-terminus is translated, which then can chaperone the N-terminal domains into a post translational mode of import. In this context Ssa1/2 can affect dual targeting by binding aconitase and decreasing the interaction between the N and C terminal domains, thereby slowing import and causing more aconitase to end up in the cytosol. In this regard the effect of Ssa1 on aconitase distribution is opposite to its effect on fumarase distribution, which we now can understand: For fumarase, the driving force for reverse translocation is folding of the whole protein; Ssa1 slows down fumarase folding which enhances translocation and more mitochondrial targeting of the enzyme. For aconitase, Ssa1 also slows down folding thereby decreasing the interaction of the C-terminal “chaperone like activity” with the “import challenging” N-terminus. This causes aconitase to be translocated slowly, possibly providing opportunity for the C-terminus “dual targeting signal” to function. This scenario is intriguing in that induction of a single molecular chaperone, Ssa1/2 (cytosolic Hsp70), may have different consequences regarding the subcellular distribution of specific proteins.

## Materials and Methods

### Growth Conditions

Cultures harboring the indicated plasmids were grown at 30 °C overnight in synthetic depleted (SD) medium containing 0.67% (w/v) yeast nitrogen base, 2% galactose/2% glucose (w/v)/2% Acetate (w/v)/ 3% Ethanol (v/v), supplemented with the appropriate amino acids (50 μg/ml). 2% agar was added for agar plates.

### α-Complementation Assay

Yeast cultures harbouring plasmids encoding the indicated α fusion proteins in combination with pω_c_ or pω_m_ were generated. Colonies were grown on 80 mg/ml X-gal plates and incubated in dark at 30 °C for 72 h.

### Pulse chase experiments and Immunoprecipitation

Yeast cultures in synthetic depleted medium with galactose (2%) were grown to *A*_600 nm_ = 1.5. Cells were harvested, washed, and resuspended in fresh galactose synthetic depleted medium without methionine. Cultures were labeled with 100μCi of [^35^S] methionine/cysteine in the presence of 20 μM CCCP and incubated for 15 minutes. Labelling and translation were stopped by the addition of excess “cold” 0.004% cysteine and 0.003% methionine, whereas 40 mM dithiothreitol (DTT) was added to re-establish the membrane potential as a consequence of the CCCP treatment. Aliquots obtained at the indicated times were placed on ice, and 10 mM sodium azide was added to the aliquots in order to stop the reaction. Labelled cultures were centrifuged and resuspended in Tris/EDTA buffer, pH 8.0, containing 1 mM phenylmethylsulfonyl fluoride, broken with glass beads for 20 min in 4 °C, and the supernatant fraction was obtained by centrifugation. Supernatant fractions were boiled in 1% SDS. Immunoprecipitation in the presence of one-fourth of the total volume of TENN buffer (200 mMTris, pH 8.0, 20 mM EDTA, 2% Nonidet P-40, 600 mMNaCl), was performed with aconitase antiserum and protein-A magnetic Dynabeads (Novex by Life technologies). Then SDS-PAGE analysis followed by visualization [BAS2000 image-analyzing system (Fuji Corp.)] and autoradiography was undertaken.

### GST Pull-down

200 ml of yeast cells were grown on YPD or selective media at 30 °C to *A*_600 nm_ = 1.5. Normalized cells were harvested, washed and transferred into 2 ml screw-capped tubes. Cells were lysed with glass beads (Sigma) and beaten with Micro Smash (TOMY) for 4 times (1 minute per time). Lysed cells were centrifuged at 10,000 g for 10 minutes at 4 °C. Supernatant was transferred to new microcentrifuge tubes. 30 µl of GST beads (GE Health Lifesciences) were washed with lysis buffer (50 mM Tris-HCL, 150 mM KCL, 1% NP-40) and resuspended in 100 µl of lysis buffer. Cell extracts were each mixed with 40 µl GST beads in 100 µl of lysis buffer and protease inhibitor cocktail (Calbiochem, 1:1000). The mixture was rotated at 4 °C for 2 hours. After incubation, the mixture was washed with lysis buffer 5 times. The mixture was added with 5X SDS loading dye (5% β-Mercaptoethanol, 0.02% Bromophenol Blue, 30% Glycerol, 10% SDS, 250 mM PH6.8 Tris-CL) and heated at 95 °C for 10 minutes.

#### Coomassie Blue Staining

GST Pull-down samples were run on a 10% SDS-PAGE gel at 150 volts for 1.5 hours. The gel was washed in water for 5 minutes and repeated 3 more times. The gel was incubated with 50 ml Coomassie G-250 Stain (Bio-Rad) in an orbital shaker for 1 hour. The gel was rinsed with water for 30 minutes.

#### In-gel-digestion (sample preparation for LC-MS/MS)

The lanes of interest were individually cut out from the gel and 1 × 1 mm pieces were washed 3 times with 1 ml dH_2_O/CH_3_CN (50:50; V:V) and then with 100 mM NH_4_HCO_3_. The gel pieces were dried in a Speedvac, brought up in 10 mM DTT and 100 mM NH_4_HCO_3_ and then were incubated at 56 °C for 1 hour. Equal volume of 50 mM iodoacetamide was added and the mixture was incubated at room temperature for 30 minutes. The pieces were washed with 100 mM NH_4_HCO_3_ and then with 20 mM NH_4_HCO_3_ containing 50% CH_3_CN. The pieces were dried in a Speedvac. 20 µl/band of 12.5 µg/ml of Trypsin Gold (Promega) in 20 mM NH_4_HCO_3_ was added. The mixture was incubated at 30 °C overnight (more than 16 hours). Equal volumes of CH_3_CN were added to the digest and the mixture was incubated at 30 °C for 30 minutes. The supernatant was retained while 50 µl of 1% formic acid was added to the gel pieces and the mixture was incubated for 20 minutes at room temperature. The supernatants were combined. The peptides were dried in a Speedvac. The pellet was resuspended with 1% formic acid and subjected to LC-MS/MS analysis.

#### LC–MS/MS analysis

The tryptic peptides were separated with an Eksigent nanolC Ultra and ChiPLC-nanoflex (Eksigent, Dublin, CA, USA) in Trap Elute configuration. The sample was loaded on a 200 μm × 0.5 mm trap column and eluted onto an analytical 75 μm × 150 mm column. Both trap and analytical columns were made of ChromXP C18-CL, 3 μm (Eksigent, Germany). Peptides were separated by a gradient formed by 2% ACN, 0.1% FA (mobile phase A) and 98% ACN, 0.1% FA (mobile phase B): 5 to 7% of mobile phase B in 0.1 min, 7 to 30% of mobile phase B in 10 min, 30 to 60% of mobile phase B in 4 min, 60 to 90% of mobile phase B in 1 min, 90 to 90% of mobile phase B in 5 min, 90 to 5% of mobile phase B in 1 min and kept at 5% of mobile phase B for 10 min, at a flow rate of 300 nl/min. The MS analysis was performed on a TripleTOF 5600 system (AB SCIEX, Foster City, CA, USA) in Information Dependent Mode. MS spectra were acquired across the mass range of 400–1250 m/z in high resolution mode (>30000) using 250 ms accumulation time per spectrum. A maximum of 10 precursors per cycle were chosen for fragmentation from each MS spectrum with 100 ms minimum accumulation time for each precursor and dynamic exclusion for 8 s. Tandem mass spectra were recorded in high sensitivity mode (resolution >15000) with rolling collision energy on adjustment. Survey-IDA Experiment, with charge state 2 to 4 which exceeds 125 cps was selected. Peptide identification was carried on the Protein Pilot 4.5 software Revision 1656 (AB SCIEX) using the Paragon database search algorithm (4.5.0.0.1654) for peptide identification and the integrated false discovery rate (FDR) analysis function. The data were searched against a database consisting of 120608 uniprot_sprot database (total 6621 entries). The search parameters were as follows: Sample Type — Identification; Cys Alkylation — MMTS; Digestion — trypsin; Special Factors — None; Species —*Saccharomyces cerevisiae*. The processing was specified as follows: ID Focus—Biological Modifications; Search Effort — Thorough; Detected Protein Threshold — 0.05 (10.0%) and competitor Error Margin (ProtScore) −2.00. Proteins identified with an unused ProtScore (Conf) ≥1.3 (95%) were accepted as positive identification.

### Modeling

Homology models of the structure of yeast aconitase (based on the solved structures of PDBs 1aco, 1c96 and 2b3y), as well as Ssa1 (based on the solved structures of PDBs 3d2f, 3qfp, 4jne and 4kbo) were generated using I-TASSER^25^. The models with the highest probability score among the provided models were selected for further investigation. Docking of Ssa1 to aconitase was performed using the PatchDock software^23^, with the structural models as input. These models were further refined with the RosettaDock local refinement algorithm^24^ to generate models with atomistic detail of the interaction. 50 refined models were generated and the ones with low-energy total Rosetta and interface scores were selected. Mutations were designed based on visual inspection of the models. Figures of structures were generated using the PyMOL software (Schroedinger Inc).

## Electronic supplementary material

Supplementary data
