# Supplementary material for: Yeast aconitase mitochondrial import is modulated by interactions of its C and N terminal domains and Ssa1/2 (Hsp70)
Source: Sci Rep. 2018 Apr 12;8:5903. doi: 10.1038/s41598-018-24068-w (PMC5897410; doi:10.1038/s41598-018-24068-w)

Yeast aconitase mitochondrial import is modulated by interactions of its C and N terminal domains and Ssa1/2 (Hsp70).

Reut Ben-menachem[1], Katherine wang[2],Orly Marcu[1], Zhang Yu[2], Teck Kwang Lim[3], Qingsong Lin[3], Ora Schueler-Furman[1] and Ophry Pines[1, 2]

[1-]Department of Microbiology and Molecular Genetics, IMRIC, Faculty of Medicine, HebrewUniversity, Jerusalem, Israel.
[2-]CREATE-NUS-HUJ Program and the Department of Microbiology, Yong Loo Lin School of Medicine, National University of Singapore, Singapore.
[3-]Department of Biological Sciences, National University of Singapore.

Figure 1B -Ben Menachem

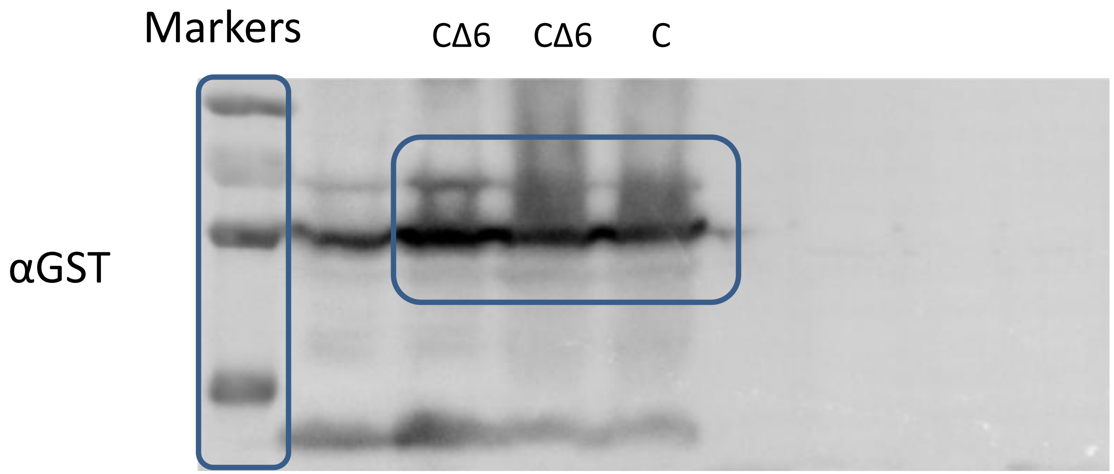

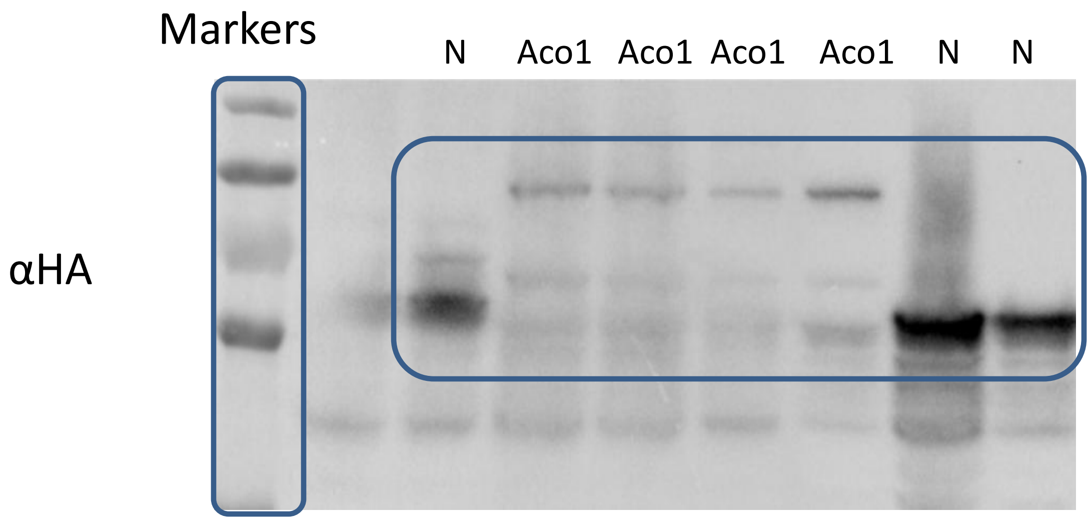

Figure 1C-Ben Menachem

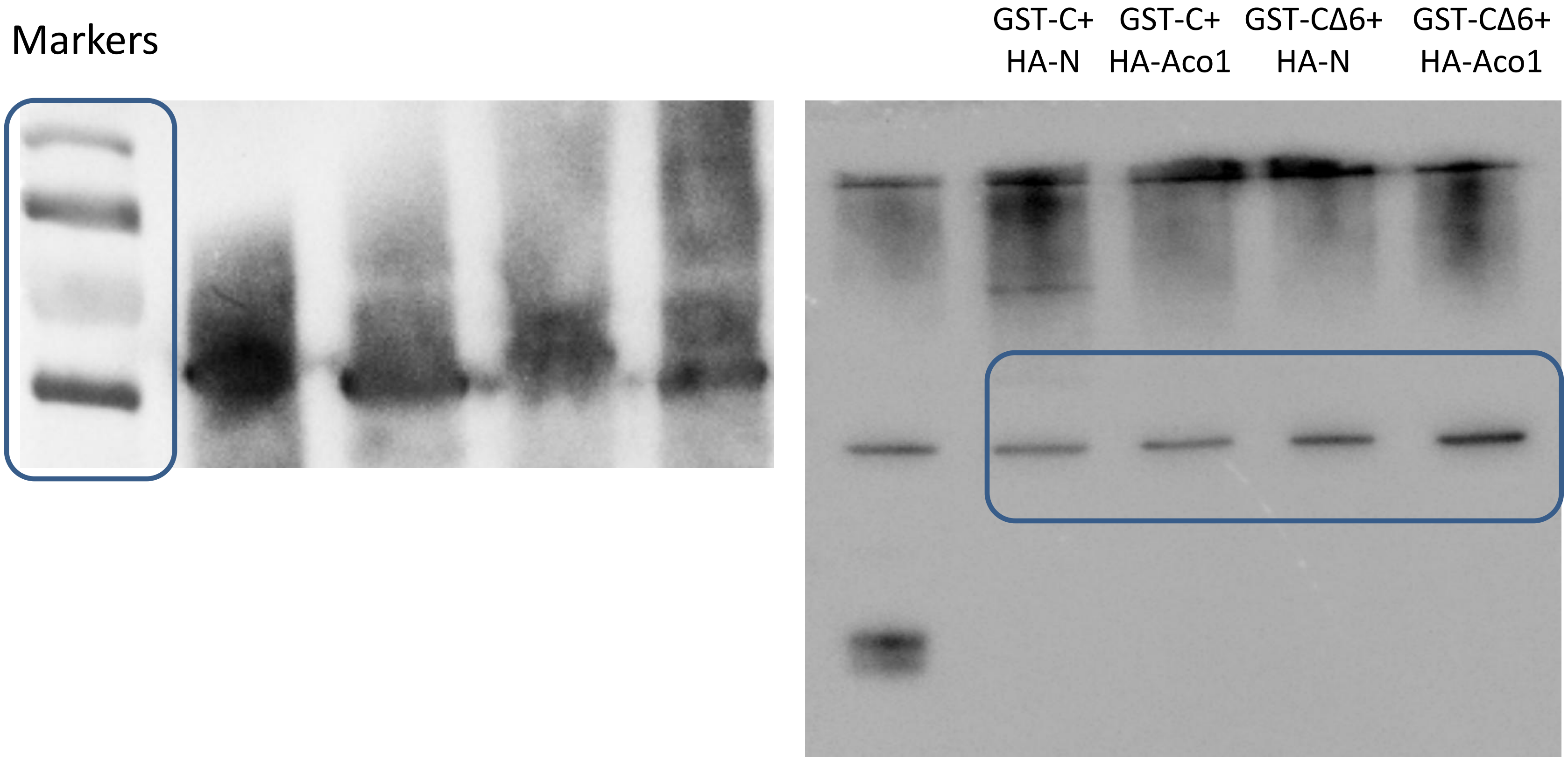

Figure 2B-Ben Menachem

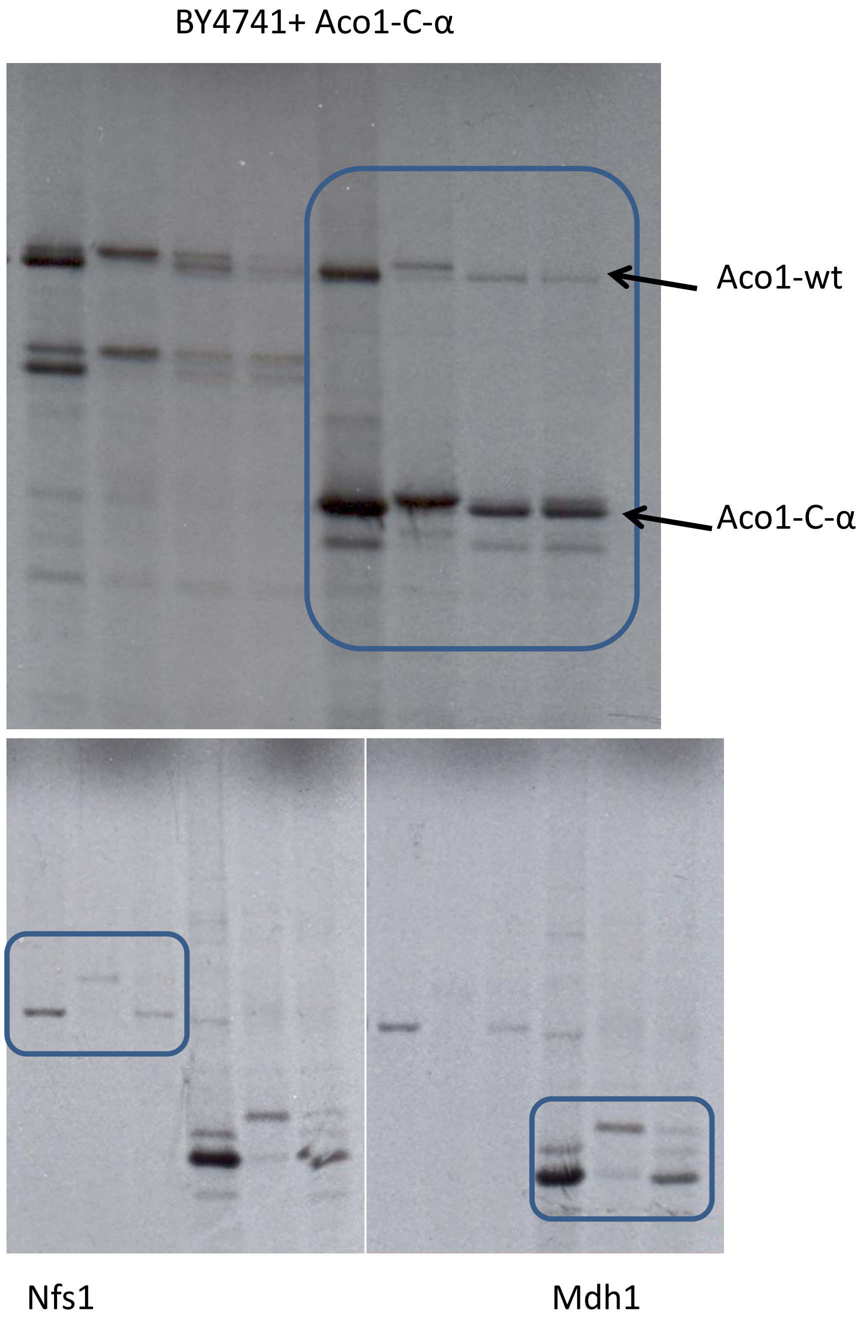

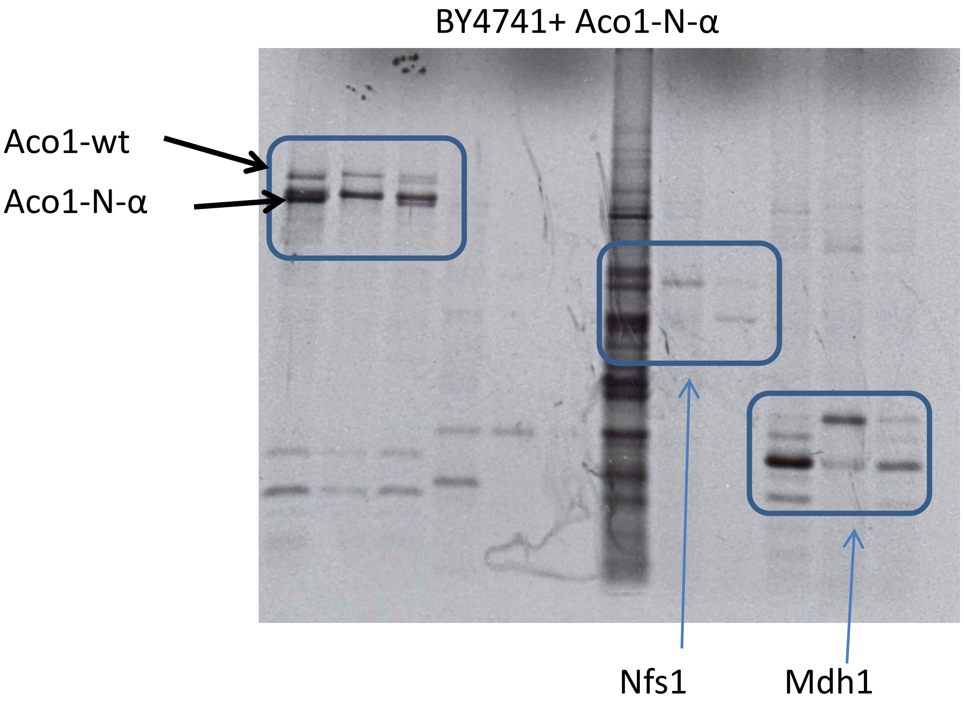

Figure 3C-Ben Menachem

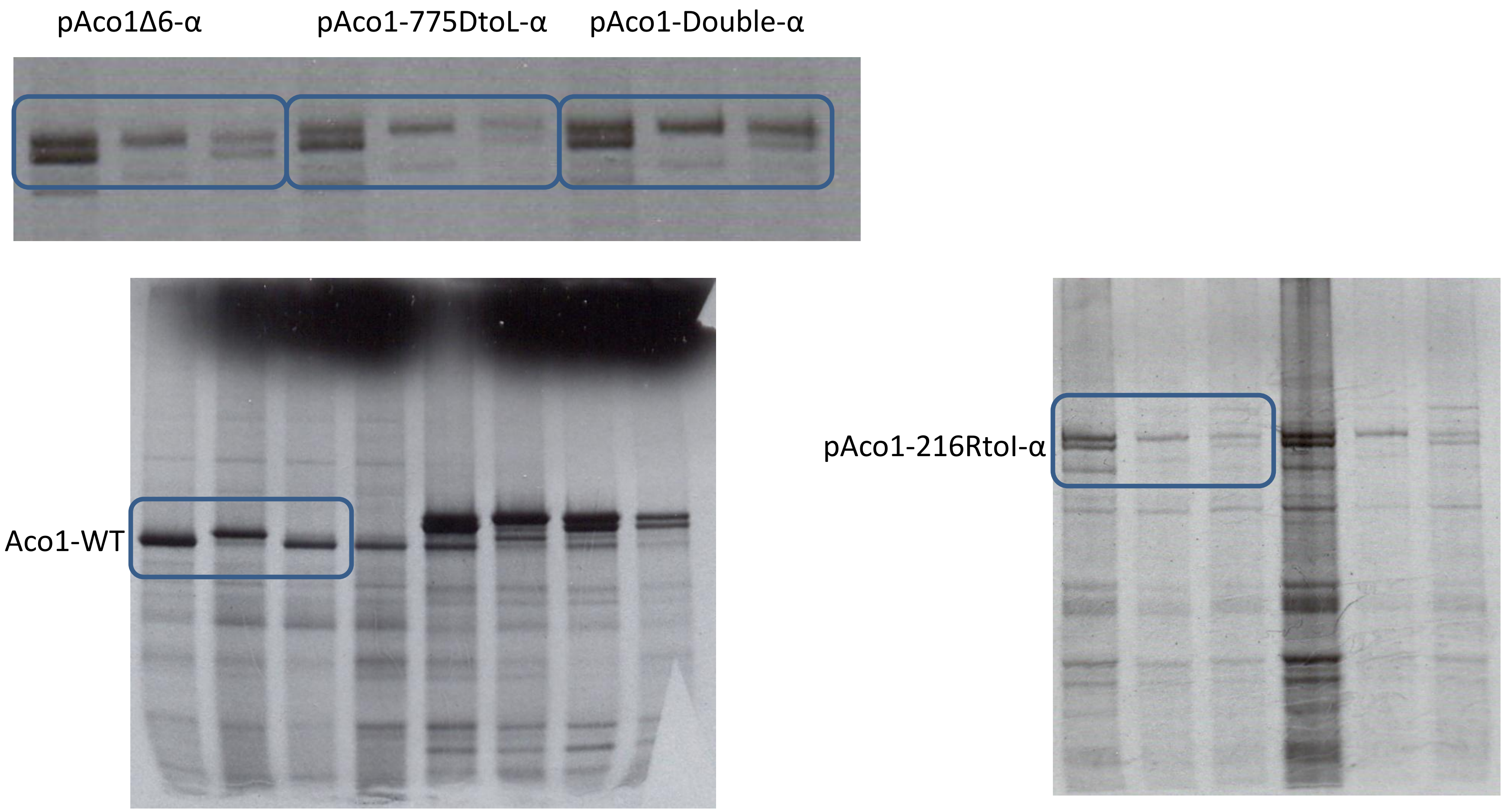

Figure 5F-Ben Menachem

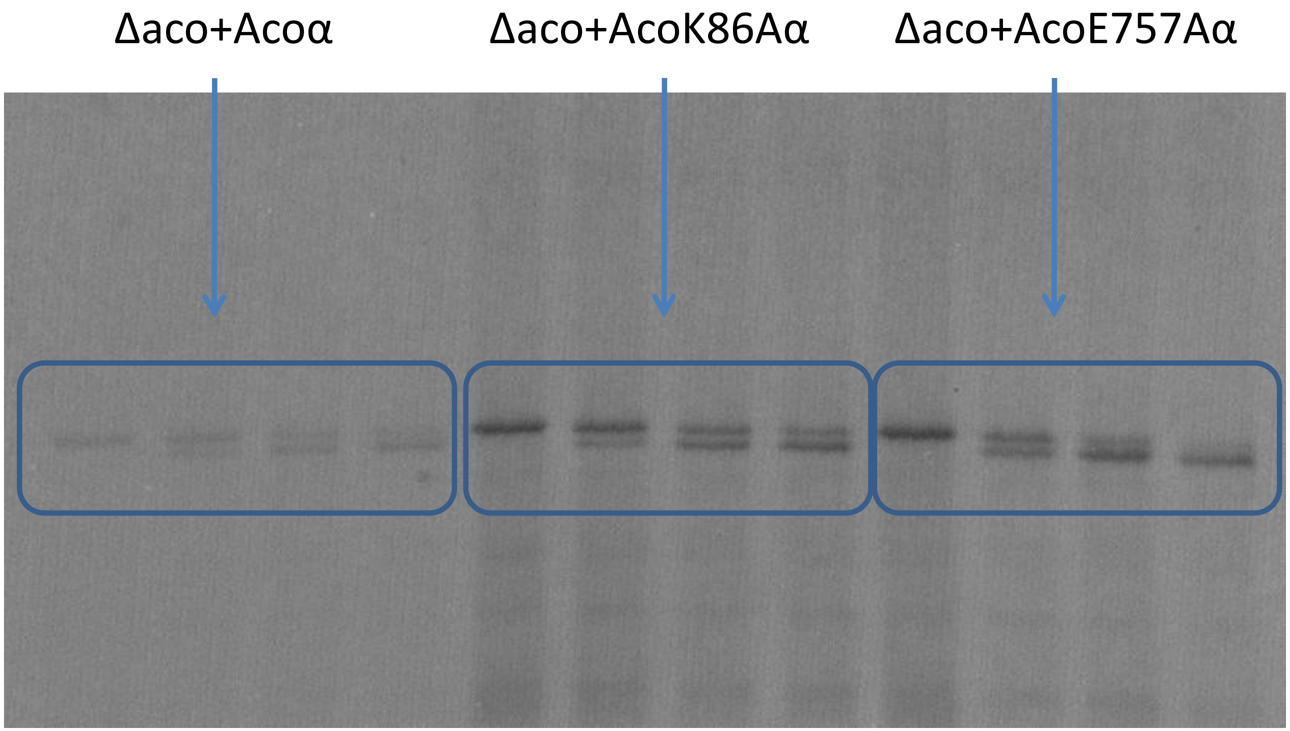

Figure 6C-Ben Menachem

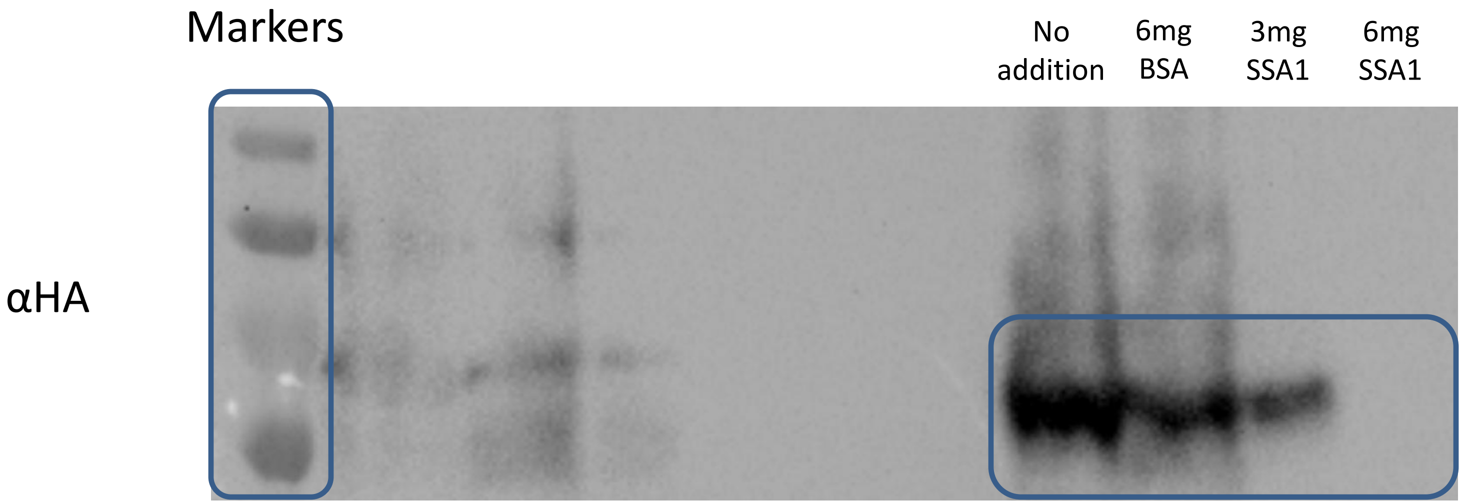

Supplement: Supplementary file 1 — Supplementary data [file 41598_2018_24068_MOESM1_ESM.pdf]
